# Building a House of Skills—A Study of Functional Health Literacy and Numeracy among Patients with Type 2 Diabetes in Hungary

**DOI:** 10.3390/ijerph18041547

**Published:** 2021-02-06

**Authors:** Andrea Klinovszky, Orsolya Papp-Zipernovszky, Norbert Buzás

**Affiliations:** 1 Department of Health Economics, University of Szeged, 6724 Szeged, Hungary; buzas.norbert@med.u-szeged.hu; 2 Department of Psychology, University of Szeged, 6722 Szeged, Hungary; papp.orsolya@psy.u-szeged.hu

**Keywords:** patients with T2DM, insulin therapy, functional health literacy, diabetes-specific numeracy skills

## Abstract

The purpose of this study is to explore functional health literacy (FHL) and numeracy skills in an insulin-treated, type 2 diabetes mellitus (T2DM) patient population, and their impact on diabetes self-care activities. A non-experimental, cross-sectional quantitative design was used for this study. The sample consisted of 102 T2DM patients on insulin therapy, including 42 males and 60 females, with a mean age of 64.75 years (*SD* = 9.180) and an average diabetes duration of 10.76 years (*SD* = 6.702). Independent variables were sociodemographic variables (e.g., age, educational level, etc.) and diabetes and health-related factors (e.g., duration of diabetes (years), the frequency of blood glucose testing/day, etc.). For this study, the participants completed the reading comprehension exercise from the Short Test of Functional Health Literacy (S-TOFHLA) and the Shortened Version of the Diabetes Numeracy Test (DNT-15), which specifically evaluates the numeracy skills of patients living with diabetes. The associations between the variables were examined with Spearman’s rank correlation. Multivariate regression analysis was performed to examine whether measured FHL skills impact diabetes self-care activities. We found that DNT-15 test (*β* = 0.174, *t*(96) = 2.412, *p* < 0.018) had significant effect on the frequency of blood glucose testing/day. Moreover, the problem areas for patients with T2DM mostly included multi-step calculations according to food label interpretations, and adequate insulin dosage based on current blood glucose levels and carbohydrate intake. The results of regression analyses and Spearman’s rank correlation indicated that limited FHL and diabetes numeracy skills not only influenced the participants’ behaviors related to self-management, but they also affected their health outcomes. Thus, besides the personalization of insulin treatment, it is indispensable to provide more precise information on different types of insulin administration and more refined educational materials based on medical nutrition therapy.

## 1. Introduction

According to the International Diabetes Federation (IDF), the number of people diagnosed with diabetes mellitus (DM) has more than tripled over the past 20 years. This report also indicated that there are currently 463 million people living with diabetes, and this number will increase to 700 million by 2045 [1]. In Europe, it is estimated that among adults aged 20 to 79, there are 59.3 million people who are diagnosed with DM, with the majority affected by type 2 diabetes mellitus (T2DM) [2]. Previous studies have shown that T2DM negatively affects patients’ quality of life and life expectancy [3], and it often places serious economic burdens on society and its healthcare systems [4].

It is well-known that patients with T2DM are required to perform daily self-care tasks such as monitoring glucose levels, following a diet plan, remembering to take antidiabetic medications and insulin at the right time, preparing the correct dosage, and performing regular physical activities, all of which can be emotionally stressful [5]. In other words, a significant number of responsibilities rests on the shoulders of patients, since appropriate disease management of T2DM is essential [6]. Thus, it has become indispensable to explore what types of specific basic skills are necessary during diabetes self-management, and how effectively patients can function in the healthcare environment [7].

In general, individuals living with diabetes are required to use multiple strategies and possess adequate skills in order to cope with their condition and reduce diabetes-related complications [8]. One of these fundamental skills is the patients’ health literacy level, which is not only a highly relevant skill for diabetes management [9,10], but it has become a critical component in improving the health outcomes of chronic illnesses (e.g., T2DM) [7]. It also plays a fundamental role in improving patients’ diabetes knowledge and competencies [11]. According to the U.S. Department of Health and Human Services (HHS), health literacy is “the degree to which individuals have the capacity to obtain, process, and understand basic health information and services needed to make appropriate health decisions [12].” The World Health Organization (WHO) adds to this definition by including cognitive and social skills as essential parts of health literacy [13].

In the literature, there are two types of health literacy: one is related to individuals’ performance and it is called performance-based health literacy, while the other one is self-assessed health literacy [14]. Martensson and Hensing point out that health literacy shows a fluctuation from situation to situation and many times depends on the environmental context of the patient [15]. Because of this, professionals often divide health literacy into three different dimensions: functional, communicative and critical health literacy [16,17,18]. One of the most widely studied dimensions is functional health literacy (FHL), which focuses on basic functional skills such as reading, writing, and numeracy [17,19]. More precisely, these skills include the ability to read and comprehend written text, complete health care forms or be able to use numeric information related to health care instructions during disease-management [10,20].

Previous empirical studies found that limited HL affects a great number of European regions, such as Italy, which is reported to have the highest number of people living with inadequate HL, and it is followed by Bulgaria, Spain and Austria [21]. It is important to mention that in 2016, Hungary joined to the European Health Literacy Project (HLS-EU) and findings from this study indicated that more than 50% of the surveyed Hungarian population had limited level of health literacy [22]. However, the aforementioned study did not focus on disease-specific FHL.

Although the number of national and international studies examining this complex phenomenon has been increasing, there are still relatively few studies on disease-specific FHL [23]. Furthermore, little is known about FHL levels and their impact on diabetes outcomes (e.g., knowledge, self-care, glycemic control) [24,25], maybe due to the complexity of the phenomenon, which is highlighted by the quantitative studies in the past 5 years. According to a meta-analysis published in 2019, HL prevalence studies among patients with T2DM were done mostly in the USA and these results mainly date back to the early 2000’s [11]. However, according to Poureslami et al. (2017), health literacy is fundamental to a successful management and prevention of chronic disease [26]. Therefore, it would be especially important to explore health literacy level in high-risk groups and its relationship with health outcomes.

### 1.1. Association between FHL and Diabetes Self-Management

Related studies have also shown that FHL levels are lower among patients with chronic diseases, compared to healthy populations [25,27,28,29]. Individuals with low FHL levels have also been shown to have less knowledge about their chronic diseases and limited self-management skills [30,31,32,33,34]. Moreover, previous research has indicated that lower FHL levels are associated with higher mortality rates, less adequate health status, more hospitalization, and lower levels of medication taken during self-management [19,24,32,33], i.e., difficulties in naming their medications and describing their indications [25].

FHL is a critical component of diabetes self-management as well. However, it is not the only variable that influences patients’ self-care skills. Both theoretical and empirical studies confirm that an individual’s self-efficacy, attitude, motivation, degree of illness severity, and social factors also impact his/her self-management behavior [35]. In addition, the connection between FHL and diabetes self-management is not straightforward, and it depends on the type of self-management behavior followed by patients [36,37]. This is especially true for individuals with diabetes and inadequate FHL, since they are recommended to follow complex treatment plans, manage visits to multiple clinicians, monitor changes in their health status, and initiate positive health behaviors [38].

Based on previous research, lower FHL levels are not necessarily associated with worse A1C levels among DM patients [10,39], but lower FHL and A1C levels are correlated with more diabetes complications [40,41]. It is also important to mention that there is a limited number of studies on understanding the pathways or potential mechanisms through which FHL influences patients’ A1C levels [42].

Although FHL affects patients’ ability to function in a healthcare system [43], this skillset could help them cope with the consequences of a chronic illness and increase their confidence during medical consultations [19]. In sum, adequate FHL can enhance patients’ understanding and awareness about a chronic disease and help facilitate their engagement in diabetes self-management [26].

### 1.2. Diabetes-Specific Numeracy Skills and Their Impact on Health Outcomes

Low diabetes-related numeracy is common among individuals with diabetes [44]. The number of clinical studies on general HL and numeracy is growing; there are much fewer still (especially in Europe) focusing on patients with specific chronic diseases such as those diagnosed with T2DM and prescribed different insulin regimes. The research by Alghodaier et al. (2017) clearly states that the influence of numeracy on the health of patients with diabetes has been investigated in a small number of studies [45]. Based on one meta-analysis it is recommended to invest the assessment of diabetes numeracy to produce more evidence on the relationship with different diabetes outcomes, including self-care activities [11]. According to previous research, numeracy refers to the ability to understand and use numbers in daily life [46] (p. 392). More precisely, in the healthcare context, numeracy not only includes the ability to interpret risk, but also the ability to estimate time and measure, think logically, and perform the mathematical skills required to solve problems and make appropriate health-related decisions [47]. In addition, Huizinga et al. (2008) emphasized that higher numeracy levels can help DM patients understand medical graphic representations [48].

Besides reading skills, numeracy skills are required in many health-related tasks such as understanding food labels, measuring medications, interpreting blood parameters, and understanding health risks and symptoms [10,31,49]. However, according to previous research, only 50% of patients with inadequate FHL and numeracy skills recognize common symptoms of hypoglycemia, while only 38% correctly respond to the need to eat when experiencing such symptoms [49]. Meanwhile, self-management of T2DM requires advanced FHL and numeracy skills [7,50,51]. They are also indispensable because patients with limited skills in reading comprehension, oral communication, and diabetes-specific numeracy will be unable to adequately participate in traditional health education and act on any instructions from healthcare providers [16]. In sum, lower levels of diabetes-specific numeracy skills not only impact patients’ health behaviors [47] but are also associated with higher A1C levels in patients with T2DM [52], which can negatively affect their adherence to recommendations and cause more complications [24,53]. Meanwhile, individuals with better mathematical skills are able to apply such knowledge, thus increasing their confidence in performing self-care tasks [49].

The purpose of this study is to explore FHL levels and numeracy skills in an insulin-treated T2DM patient population are related to diabetes, and they impact on diabetes therapy. In addition, diabetes and health-related variables and diabetes self-care activities associated with FHL and numeracy are examined.

## 2. Materials and Methods

### 2.1. Research Design

A non-experimental, cross-sectional quantitative research design was used in this study, with stratified convenience sampling method.

### 2.2. Data Collection

This research was conducted between January and March 2020. The participants were recruited with the help of three diabetes associations in Csongrád County, Hungary. The members of the diabetes associations were selected with stratified convenience sampling. They were notified about the research in advance by the president of the association. Research subjects who satisfied the requirements and expressed their willingness to participate in the study came to the study location at a time which was agreed upon in advance. Before the interview, research subjects were notified in detail about the purpose of the study and that their information would be kept strictly confidential and anonymous. The patients were also informed about their right to discontinue the interview and that there would be no consequences whatsoever to their participation. The response rate was 78.46% (out of 130 patients contacted, 102 were interviewed). All of the participants were involved on a voluntary basis and their written informed consent was obtained in accordance with the Declaration of Helsinki. This study was approved by the University of Szeged Human Investigation Review Board (Approval No. 4639).

### 2.3. Study Sample and Inclusion/Exclusion Criteria

The inclusion criteria for the participants were as follows: 18 to 89 years of age; diagnosed with T2DM; use of insulin therapy; voluntary participation in the study. Regarding the exclusion criteria, cerebral stroke affecting cognitive processes or previous head injuries were listed.

### 2.4. Study Measurements

#### 2.4.1. Short Test of Functional Health Literacy (S-TOFHLA)

The Short Test of Functional Health Literacy (S-TOFHLA) normally includes reading comprehension and numeracy tasks. However, since the goal of this study was to examine diabetes-related health literacy, a diabetes-specific numeracy test was included, instead of the original calculation exercises. The other reason why the S-TOFHLA’s computational tasks were not used was because the numeracy portion of the Hungarian version showed low internal reliability. During the S-TOFHLA’s reading comprehension exercise, the participants were asked to read medical information on abdominal x-rays (3rd grade comprehension level; 16 questions) and a text related to health insurance contracts (9th grade comprehension level; 20 questions), after which they were asked to complete certain sentences by choosing one out of four options. Although the total time to complete the exercise was seven minutes, the participants were not informed about this time constraint. Thus, the interviewer stopped the exercise when the time limit was reached. Overall, each correct answer was worth 1 point, while any questions that were incorrect, left blank, or answered after the time limit were worth 0 points. The maximum score for the reading comprehension exercise was 36 points. Based on the scores from the exercise, 0–16 points indicated inadequate health literacy, 17–22 points indicated marginal health literacy, and 23–36 points indicated adequate health literacy [27,34].

#### 2.4.2. Shortened Version of the Diabetes Numeracy Test (DNT-15)

In order to better understand numeracy in T2DM, a shortened version of the Diabetes Numeracy Test (DNT-15) was used. In general, the DNT-15 evaluates the numeracy skills of diabetes patients, and it consists of 15 questions in five domains: nutrition (three items); exercise (one item); blood glucose monitoring (three item); oral medications (one item); insulin administration (seven items). The estimated administration time of the DNT-15 is 15 to 20 min. This scale also covers different types of mathematical problems such as addition, multiplication/division, fractions, multi-step mathematics, and numeration/number hierarchy [49].

#### 2.4.3. Principles of the DNT-15, Including Interpretation and Measurement Characteristics

To the best of the authors’ knowledge, diabetes-specific numeracy skills have not been studied in Hungary among T2DM patients on insulin therapy. Since the S-TOFHLA’s numeracy tasks in the Hungarian version showed low reliability, and in an effort to use a test that effectively measures diabetes-specific numeracy skills, the decision was made to translate one of the most common instruments for measuring diabetes-specific numeracy skills, i.e., the DNT-15. The test was translated from English into Hungarian by applying standardized translation methods, such as back-translation and cultural adaptations, which are essential steps before implementing an instrument in research [54,55,56,57]. Before using the translated version of the DNT-15 instrument on a larger sample, a pretest was conducted with 10 independent respondents, who completed the test in order to determine if the tasks were comprehensible and if there were any legitimate comments about the test questions. It is important to note that although the Hungarian version of the DNT-15 test had adequate reliability, the results of this study should be interpreted with caution, mainly due to the fact that the Hungarian version of the DNT-15 has yet to be validated.

### 2.5. Additional Independent Variables

Overall, the analysis included two groups of independent variables. The first group included sociodemographic variables such as age, gender, labor market status, education level, and whether the participant possessed healthcare education. The second group of independent variables included diabetes and health-related factors such as duration of diabetes (years), duration of insulin treatment (years), daily frequency of self-monitoring blood glucose levels, self-assessed three-month A1C level, frequency of insulin administration per day, existence of diabetes complications, and type of diabetes therapy (e.g., only insulin/insulin + antidiabetic drugs/insulin + lifestyle therapy/insulin + antidiabetic drugs + lifestyle therapy).

### 2.6. Statistical Analysis

The statistical analysis was performed by using IBM SPSS Statistics for Windows 22 (IBM Corporation, Armonk, NY, USA). After the preparation of the statistical data, any missing values were detected and the outliers in the sample were appropriately checked. In addition, the Kolmogorov-Smirnov Goodness-of-Fit Test was used to assess the data distribution. For the purpose of obtaining correct results from the statistical analysis, it was important to note the following: the analysis was conducted on a smaller sample; there was data with variables that also included ordinal scales; the conditions for normality deviation were not always met. Thus, non-parametric tests were performed. In order to describe the population’s characteristics and provide basic information about the variables in the dataset, descriptive statistics were used. Internal consistency and reliability of the measurements were tested with Cronbach’s alpha and Kuder–Richardson’s reliability coefficient, while the associations between the variables were examined with Spearman’s rank correlation. Any differences between the groups were then examined with the Mann–Whitney test. In order to suppress any type 1 errors, the significance value of the tests was examined using Bonferroni correction, while the dependent variables with ordinal values were examined using the Kruskal–Wallis test. Multivariate regression analysis was performed to examine whether measured FHL and diabetes numeracy skills impact diabetes self-care activities.

## 3. Results

### 3.1. Descriptive Statistics

The final sample consisted of 102 patients (41.2% male and 58.8% female) diagnosed with T2DM for an average of 10.76 years (*SD* = 6.702). The mean age of the sample was 64.75 years (*SD* = 9.180; range 37–85 years). The majority of the participants had high school (*N* = 58) education, while 29 participants completed primary school and 15 possessed a university degree. The average duration of insulin treatment was 6.59 years (*SD* = 5.098), while the majority (45.1%) of the participants reported administering insulin four times per day. It is important to note that most of the patients (67.6%) measured their blood glucose levels more than twice per day, while 73.5% had diabetes complications. In terms of the latter, the distribution was as follows: vision impairment (retinopathy): 52% (*N* = 53); cardiovascular disease: 44.1% (*N* = 45); kidney failure (nephropathy): 7.8% (*N* = 8); nerve damage (neuropathy): 45.1% (*N* = 46); lower limb amputation: 6.9% (*N* = 7) (Table 1).

### 3.2. Average Score of the Correct Responses on the S-TOFHLA and DNT-15, and the Internal Consistency of the Measurements

In the S-TOFHLA’s reading comprehension task, participants were asked to read medical information on abdominal x-rays (3rd grade comprehension level; 16 questions) and a text related to health insurance contracts (9th grade comprehension level; 20 questions). Average score of correct responses for task “A” (3rd grade comprehension level) was 78.5%, while average score of correct responses for task “B” (9th grade comprehension level) was 56.4%. Because of the 7-min timeframe, the rate of correct responses for item 6 in task “B” was lower. The average score on the S-TOFHLA was 23.78 points (*SD* = 10.084), while the 36-item scale included a Cronbach’s alpha of 0.957, indicating high reliability. Based on the results, 27.7% of the participants (28 patients) had inadequate FHL, 6.9% (7 patients) had marginal FHL, and 65.3% (66 patients) had adequate FHL (Table 2).

As for the DNT-15, it showed good internal reliability (KR-20 = 0.85), with an average score of 7.51 (*SD* = 3.509). The results also indicated that the problem areas for the participants included food label interpretation and items that required multi-step calculations such as calculating insulin dosage based on current blood glucose levels and carbohydrate intake.

### 3.3. Associations between the S-TOFHLA Scores and the Diabetes and Health-Related Factors

The associations between the S-TOFHLA scores and the diabetes and health-related variables were analyzed with Spearman’s rank correlation. The results showed a significant negative, weak relationship between the S-TOFHLA scores and the years of diabetes duration (*r_s_* (99) = −0.198, *p* = 0.048), with a significance level of 0.05.

As for the relationship between the S-TOFHLA scores and the frequency of self-monitoring blood glucose levels per day, the results showed a significant positive, weak correlation (*r_s_* (99) = 0.370, *p* < 0.001), with a significance level of 0.01. However, there was no significant relationship between the S-TOFHLA test scores, the self-assessed three-month A1C level (*r_s_* (99) = −0.189, *p* = 0.058), and the patients’ daily insulin administration (*r_s_* (99) = −0.175, *p* = 0.081) (Table 3).

### 3.4. Associations between the DNT-15 Scores and the Patients’ Diabetes and Health-Related Factors

The associations between the DNT-15 scores and the patients’ diabetes and health-related variables were also analyzed with Spearman’s rank correlation. The results showed a significant positive, weak correlation between the DNT-15 scores and the patients’ daily insulin administration (*r_s_* (100) = 0.272, *p* = 0.006), while the relationship between the DNT-15 scores and the frequency of self-monitoring blood glucose levels per day indicated a significant positive, moderate correlation (*r*_s_ (100) = 0.473, *p* < 0.001). Based on the findings, the higher scores on the DNT-15 test showed no statistically significant association with the duration of diabetes (*r_s_* (100) = −0.108, *p* = 0.282), and the self-assessed three-month A1C level (*r_s_* (100) = −0.086, *p* = 0.392) (Table 3).

### 3.5. Associations between the Number of Diabetes Complications and the DNT-15 Scores

In order to determine the differences between the various groups of patients with diabetes complications and their DNT-15 scores, the Kruskal–Wallis test was used. Based on the statistical analysis, the Kruskal–Wallis test was significant (*H (4)* = 12.690, *p* = 0.013). To determine which groups significantly differed, the Mann–Whitney test was used. In addition, the Bonferroni corrected significance level was 0.0125. Based on the findings, the participants who performed significantly better on the DNT-15 had significantly fewer diabetes complications.

### 3.6. The Effect of Sociodemographic Variables on the Results of the S-TOFHLA Test Measuring Functional Health Literacy

In order to reveal which sociodemographic variables have the most powerful effect on the results of the S-TOFHLA test, we performed a Multivariate Regression Analysis. The dependent variable was the total score on the S-TOFHLA, and the independent variables were age, education level and the possession of healthcare education. The model proved to be significant during the multivariate regression analysis (*F*(2,98) = 47.438, *MSE* = 0.518, *p* < 0.001). Its explanatory power was R^2^ = 49.2% in the sample, R^2^_Adj_ = 48.2% in the population. From the sociodemographic variables, age had a greater effect on the S-TOFHLA results (*β* = −0.415, *t*(98) = −5.712, *p* < 0.001), but the effect of the education level was also significant (*β* = 0.513, *t*(98) = 7.059, *p* < 0.001).

### 3.7. The Effect of Sociodemographic Variables on the Results of the DNT-15 Test Measuring Numeracy Skills

We used a Multivariate Regression Analysis in order to reveal which sociodemographic variables have the greatest effect on the results of the DNT-15 test. During the analysis, we used standardized values. The dependent variable was the total score on the DNT-15, and the independent variables were age, education level and the possession of healthcare education. The model proved to be significant during the multivariate regression analysis (*F*(2,99) = 26.765, *MSE* = 0.662, *p* < 0.001). Its explanatory power was R^2^ = 35.1% in the sample and R^2^_Adj_ = 33.8% in the population. From the sociodemographic variables, age had a greater effect on the DNT-15 results (*β* = −0.380, *t*(99) = −4.657, *p* < 0.001), but the effect of the education level was also significant (*β* = 0.408, *t*(99) = 4.997, *p* < 0.001).

### 3.8. The Broadest Multivariate Regression Model Including Control Variables

In order to investigate the effects of FHL on managing diabetes we examined the daily frequency of self-monitoring blood glucose levels as a dependent variable. The model proved to be significant (*F*(1,99) = 17.430, *MSE* = 0.867, *p* < 0.001). Its explanatory power was R^2^ = 15% in the sample and R^2^_Adj_ = 14.1% in the population. The effect of the total scores on S-TOFHLA was significant (*β* = 0.387, *t*(99) = 4.175, *p* < 0.001). After that we added the DNT-15 test results to the model as a control variable. The model proved to be significant (*F*(1,99) = 27.486, *MSE* = 0.799, *p* < 0.001). Its explanatory power was R^2^ = 21.7% in the sample and R^2^_Adj_ = 20.9% in the population. The effect of the total scores on S-TOFHLA was not significant to the dependent variable in the presence of the control variable; since according to the regression analysis, the total score on the DNT-15 had a greater effect on the frequency of blood glucose testing/day variable (*β* = 0.466, *t*(98) = 5.243, *p* < 0.001).

We examined which sociodemographic, diabetes and health-related variables can predict most effectively the frequency of blood glucose testing/day besides the total scores on the two FHL tests (S-TOFHLA and DNT-15). The independent variables were the following: age, education level, possession of the healthcare education, diabetes duration (years), duration of insulin treatment (years), self-assessed three-month A1C level (mmol/L) and frequency of insulin administration per day. The regression model proved to be significant (*F*(4,96) = 41.920, *MSE* = 0.383, *p* < 0.001). Its explanatory power was R^2^ = 63.6% in the sample and R^2^_Adj_ = 62.1% in the population. According to the results, the frequency of insulin administration per day (*β* = 0.609, *t*(96) = 9.475, *p* < 0.001), the education level (*β* = 0.324, *t*(96) = 4.365, *p* < 0.001), the possession of healthcare education (*β* = 0.171, *t*(96) = 2.561, *p* < 0.012) and the total scores on the DNT-15 test (*β* = 0.174, *t*(96) = 2.412, *p* < 0.018) had an effect on the frequency of blood glucose testing/day.

## 4. Discussion

This cross-sectional study revealed that 34.6% of the patients with T2DM had inadequate/marginal reading and comprehension levels. This finding is in line with the study by Papp-Zipernovszky et al. (2016), which was the first research in Hungary that compared the scores of individuals with chronic illness to those of a healthy population and found that chronic patients had significantly lower FHL levels. Related studies by Agad Hashim et al. (2020), Abdullah et al. (2019) and Van der Heide et al. (2014) also support the aforementioned result in which the presence of type 2 diabetes is in association with the individuals’ FHL levels [35,58,59]. In addition, the results of the present study are consistent with the data of Cavanaugh et al. (2011), in which lower FHL levels among diabetes patients were between 15 and 40%, depending on the sample. It seems the Hungarian insulin-user T2DM population belongs to the worse performing range.

The results of the DNT-15 showed that the problem areas for the participants included food label interpretation and items that required multi-step calculations such as calculating insulin dosage based on current blood glucose levels and carbohydrate intake. These findings suggest that the majority of patients with T2DM provides lower average score of correct answers on these tasks within the DNT-15 test. In addition, most of these patients may also experience difficulties during nutrition therapy and when measuring the correct insulin dosage, as part of their diabetes management. This may be due to a number of factors. First, there are currently no diabetes-specific FHL and numeracy guidelines. It is also important to note that since the DNT-15 has yet to be validated on a Hungarian sample, the results should be interpreted with caution. Second, the course of diabetes varies from individual to individual, and that social and environmental factors play an important role during chronic disease management. Third, the authors’ previous study found that the majority of patients with T2DM already had a lower engagement level in lifestyle therapy. Thus, they felt less motivated to understand the nutritional value of foods, and they did not apply these competencies during insulin administration [60]. Fourth, diabetes education in Hungary does not emphasize the acquisition of the aforementioned competencies. Finally, due to the nature of the tasks, it may have caused anxiety among the participants, which, in turn, may have negatively affected their working memory and increased the number of errors performed during the tasks [31].

The associations between the S-TOFHLA scores and the years of diabetes duration showed a significant negative, weak relationship. This result suggests that patients diagnosed with T2DM for a longer period of time performed worse on the S-TOFHLA. These findings are consistent with the observations of previous research [35,51]. A potential explanation may be that diabetes negatively affects the cognitive performance of patients with DM [32]. In addition, some studies indicated that individuals who have had diabetes for more than 10 years have significantly lower FHL levels [35]. This finding is in line with the study by Yamashita and Kart, which found that some health-literate patients are overwhelmed by long-term diabetes self-management, and as a result, they feel less motivated to concentrate on the required tasks [51].

According to the findings of the present study, the relationship between the S-TOFHLA scores and the frequency of self-monitoring blood glucose levels per day showed a significant positive weak correlation. This indicates that the individuals who performed better on the S-TOFHLA reading and comprehension tasks measured their blood glucose levels more frequently. This finding is in line with previous conclusions about the associations between different FHL measurements and diabetes self-care behaviors [23,27,28,29,31]. More precisely, patients with adequate FHL skills have more knowledge about their disease. Hence, they are more conscious about their diabetes self-care activities.

A significant positive weak correlation between the DNT-15 scores and the patients’ daily insulin administration was also found. This suggests that patients with T2DM who performed better on the DNT-15 injected insulin more frequently. This finding can be explained by the fact that patients in intensive insulin therapy generally require advanced diabetes-specific numeracy skills, i.e., interpreting blood glucose meter data, administering medication dosages, and following nutritional recommendations [31,48,61]. Moreover, a significant positive, moderate correlation was found between the DNT-15 results and the frequency of self-monitoring of blood glucose levels per day. This supports the fact that numeracy, as a component of FHL, plays an important role in diabetes self-management [47].

Based on the results, the higher scores on the DNT-15 test showed no statistically significant association with the duration of diabetes. This finding is consistent with the observations of previous studies [45,47]. However, it is possible that the diabetes-specific numeracy skills do not show an explicit connection with the time of diabetes diagnosis, since such skills may be more related to an individual’s self-efficacy and self-management behavior [62]. In other words, it may be interpreted as part of the overall learning process [60]. Yet, it is feasible that patients in Hungary require more education of these skills, due to previous paternalistic viewpoints [63]. More precisely, these paternalistic viewpoints imply the fact that the physicians make decisions based on what they discern to be in the patient’s best interests and neglecting the involvement of patients in therapy [64].

In this study, the DNT-15 scores showed no statistically significant association with the patients’ self-assessed three-month A1C level. This finding supports the outcomes of previous research [52,61,65,66], which is not surprising since glycemic control is most likely influenced by a large number of heterogeneous social and biological determinants [61]. This also indicates that the impact of other variables is equally important when explaining the background of A1C level changes.

In the present study, the participants who performed better on the DNT-15 had significantly fewer diabetes complications. This result is similar to the findings of Chakkalakal et al. (2017) and Rothman et al. (2008) [29,31]. It is widely shown that adequate FHL and numeracy skills influence the health outcomes of patients, and they also improve an individual’s self-efficacy and self-care behavior. In light of the results of the DNT-15, it could be stated that diabetes patients with good numeracy skills are more likely to have greater confidence in performing self-care tasks [61]. Consequently, the number of hypoglycemic episodes and diabetes complications should also decrease [48].

We found that two the sociodemographic variables, age and education level had a greater impact on the results of the S-TOFHLA and the DNT-15 tests. These findings are similar to the findings of Gomes et al. (2020), Abdullah et al. (2019) and Yeh et al. (2019) who emphasize that age and education level are important predictors of the individuals’ FHL performance [25,59,67]. It could also be stated that patients with T2DM who are older and have a lower education level may require more attention from health care providers and diabetes educators.

Our research also tried to find answers to the questions whether FHL and diabetes-numeracy levels with sociodemographic, diabetes-specific and health-related variables impact patients’ diabetes self-care activities. During our analyses, the effect of the total scores of S-TOFHLA was significant, still we added the DNT-15 test results to the model as a control variable. When S-TOFHLA and DNT-15 were both part of the regression analysis, the total score of the DNT-15 had a greater effect on the frequency of blood glucose testing per day, which is one of the most important diabetes self-care activities during diabetes self-management [42]. Previous studies by Yarmohammadi et al. (2019), Lee et al. (2016), van der Heide et al. (2014), [35,39,42] showed similarities with the current findings and they also emphasized that self-management of T2DM requires advanced FHL and numeracy skills. These results can be interpreted by the fact that patients with a higher health literacy level check their blood-glucose levels more frequently, which is vital for patients under insulin therapy. Scores on the DNT-15 test seem to have a greater impact on diabetes self-management because diabetes numeracy does not only include the ability to interpret risk, but also to interpret blood parameters, understand food labels and measure medications. Diabetes numeracy also helps patients solve problems and make appropriate health-related decisions. This is, in practice, much more closely related to daily diabetes self-management tasks than reading and comprehension. All in all, it is conceivable that diabetes numeracy as a component of FHL skills has an important effect on patients’ diabetes self-care.

In addition to these analyses, it was also examined which sociodemographic, diabetes and health-related variables besides the total scores on the two FHL tests (S-TOFHLA and DNT-15) could have a greater impact on the frequency of blood glucose testing per day among patients with T2DM. The findings showed that the frequency of blood glucose testing per day is mostly predicted by the frequency of insulin administration per day, education level, possession of healthcare education and the total score on the DNT-15 test. The results can be explained by a fact that self-care activities, such as the frequency of blood glucose testing and the frequency of insulin administration, many times go hand in hand and both are important parts of T2DM therapy. The findings of the study are in line with the previous researches according to which diabetes therapy and self-management can be successful only if patients follow complex treatment plans of diabetes therapy [25,32,68]. In sum, it seems that one of the important key factors for successful diabetes self-care are diabetes numeracy skills which could help patients to cope with the consequences of a chronic illness, increase patients’ understanding and awareness of their chronic disease and help facilitate their engagement in diabetes self-management [19,26].

### Limitations and Future Directions

There are several limitations in this study worth noting. First, this research was based on a cross-sectional design, and it may require a larger sample size to provide higher accuracy. Second, the participants were involved on a voluntary basis. Thus, it is possible that those who were less motivated and had more problematic FHL and numeracy skills were not included. Third, all of the participants were members of various diabetes associations. Hence, they were possibly more informed and had higher FHL and numeracy skills, compared to other patients. The fourth limitation is that the DNT-15 test has not yet been validated on a Hungarian sample, so it would be recommended to do the validation procedure and only then formulate conclusions. According to the information given by patients, it is important to state that the majority of respondents self-reported a three-month A1C level. It is conceivable that some individuals did not reveal their real three-month A1C values. Our study can be considered innovative among scientific surveys because FHL and numeracy are less explored in patients diagnosed with T2DM. Furthermore, these T2DM-specific results are among first ones measured in Central-Eastern European regions, such as Hungary. It would be advisable to carry out group comparisons in the future and test these factors with patients who are not motivated to join associations. All in all, one of the most important future steps could be to carry out a longitudinal investigation which could help to explore patterns of change in and the dynamics of patients’ behavior, and also gain more insights into the cause and effect processes. Overall, it could be stated that the results obtained may also be useful in the preparation of future diabetes education materials and programs. Overall, the findings of this study could serve as a helpful reference in the preparation of future diabetes education materials and programs.

## 5. Conclusions

The study explored FHL levels and numeracy skills in an insulin-treated T2DM patient population, and their impact on diabetes self-care activities. Based on the findings, FHL and diabetes-specific quantitative skills influenced the patients’ health outcomes (e.g., the existence of diabetes-related complications) and impacted their diabetes self-care activities such as the daily blood glucose testing or daily insulin administration. This implies that diabetes numeracy skills also influence patients’ behavior towards diabetes treatment. The results also showed that the problem areas for patients with T2DM included multi-step calculations such as food label interpretation and adequate insulin dosage based on current blood glucose levels and carbohydrate intake. Therefore, besides the personalization of insulin treatment, it is indispensable to provide more precise information on different types of insulin administration and more refined educational materials based on medical nutrition therapy.

## Figures and Tables

**Table 1:** Sociodemographic characteristics of the sample and descriptive statistics of the study variables.

Sociodemographic Factors	*N* = 102
**Gender**	
Male, n (%)	42 (41.2%)
Female, n (%)	60 (58.8%)
**Age (years), mean (SD), range**	64.75 (9.180), 37−85
37−49	8 (7.8%)
50−59	14 (13.8%)
60−69	49 (48%)
70−79	27 (26.5%)
80−85	4 (3.9%)
**Education level**	
Primary school	29 (28.4%)
High school	58 (56.8%)
University	15 (14.7%)
**Diabetes complications**	
Vision impairment	53 (52%)
Cardiovascular disease	45 (44.1%)
Kidney failure	8 (7.8%)
Nerve damage	46 (45.1%)
Lower limb amputation	7 (6.9%)
**Duration of diabetes (years) mean (*SD*)**	11 (6.702)
**Duration of insulin injection (years) mean (*SD*)**	7 (5.098)
**Number of insulin injections/day mean (*SD*)**	3 (1.016)
Once	8 (7.8%)
Twice per day	23 (22.5%)
Three times per day	24 (23.5%)
Four times per day	46 (45.1%)
More than four times per day	1 (1%)
**Frequency of blood glucose testing/day** **mean (*SD*)**	3 (1.320)

Percentage based on the number of participants per item. Abbreviations: *SD* = standard deviation.

**Table 2:** The percentage distribution of the sample’s FHL levels in the S-TOFHLA.

S-TOFHLA Scores	*N* (%)
Inadequate functional health literacy (0−16)	28 (27.7%)
Marginal functional health literacy (17−22)	7 (6.9%)
Adequate functional health literacy (23−36)	66 (65.3%)

**Table 3:** The relationship between the S-TOFHLA and the DNT-15 scores, diabetes and health-related variables and Spearman’s rank correlation values.

	S-TOFHLA	DNT-15
Duration of diabetes	−0.198 *	−0.108
Frequency of blood glucose testing/day	0.370 **	0.473 **
Self-assessed three-month A1C level	−0.189	−0.086
Daily insulin administration	−0.175	0.272 *

Notes: * *p* < 0.05; ** *p* < 0.01.

## Data Availability

The data presented in this study are available upon request from the corresponding author. The data are not publicly available due to participants of this study not agreeing for their data to be shared publicly and because health data belong to the category of ‘sensitive data’.
